# Jejunal stenosis as a sequela after laparoscopic sleeve gastrectomy for morbid obesity: a case series

**DOI:** 10.1007/s13304-023-01545-0

**Published:** 2023-06-06

**Authors:** Hosam Hamed, Mahmoud Abdelwahab Ali, El-Sayed Abou El-Magd

**Affiliations:** Department of General Surgery, Faculty of Medicine, Gastrointestinal Surgical Center GISC, Mansoura University, Gehan Street, Mansoura, 35511 Al Dakahlia Governorate Egypt

**Keywords:** Laparoscopic sleeve gastrectomy, Jejunal stenosis, Porto-mesentric venous thrombosis, Complication, Ischamic bowel stricture, Bariatric surgery

## Abstract

Porto-mesenteric venous thrombosis (PMVT) is a rare complication that is encountered in less than 1% of patients following laparoscopic sleeve gastrectomy (LSG). This condition could be conservatively managed in stable patients with no evidence of peritonitis or bowel wall ischemia. Nonetheless, conservative management may be followed by ischemic small bowel stricture, which is poorly reported in the literature. Herein, we present our experience regarding three patients who presented with manifestations of jejunal stricture after initial successful conservative management of PMVT. Retrospective analysis of patients who developed jejunal stenosis as a sequela after LSG**.** The three included patients had undergone LSG with an uneventful post-operative course. All of them developed PMVT that was conservatively managed mainly by anticoagulation. After they were discharged, all of them returned with manifestations of upper bowel obstruction. Upper gastrointestinal series and abdominal computed tomography confirmed the diagnosis of jejunal stricture. The three patients were explored via laparoscopy, and resection anastomosis of the stenosed segment was performed. Bariatric surgeons should be aware of the association between PMVT, following LSG, and ischemic bowel strictures. That should help in the rapid diagnosis of the rare and difficult entity.

## Introduction

Morbid obesity has been a great health burden for developed and developing countries [1]. Laparoscopic sleeve gastrectomy (LSG) is one of the most popular bariatric procedures performed, because of its technical simplicity and excellent weight loss outcomes when compared to other procedures like Roux en y gastric bypass [2].

Despite the previous advantages, LSG has its own morbidity. The most common post-operative complications include leakage, hemorrhage, and gastric stenosis [3–5].

Another notoriously reported complication is porto-mesenteric venous thrombosis (PMVT) which could be encountered in 0.3–1% of these cases [6–8]. PMVT could be managed by medical treatment (anticoagulation) or require surgical intervention depending on the clinical presentation of the patient [9]. Delayed management could lead to bowel ischemia, infarction [10], and even liver cell failure requiring subsequent liver transplantation [11].

Previous series and case reports have reported the incidence of ischemic bowel strictures following PMVT [12–14]. Nonetheless, no previous study has reported the incidence of this rare complication in LSG patients after developing PMVT.

Herein, we present our experience regarding three patients who presented with manifestations of small bowel stricture after initial successful conservative management of PMVT. These patients of jejunal stenosis after LSG had been referred to our center for surgical management.

## Case presentation

### Case 1

A 46-year-old woman underwent LSG for obesity (initial BMI = 44.45 kg/m^2^) two years ago, and his post-operative course was uneventful.

Interestingly, antithrombotic prophylaxis was performed, using mechanical compression during surgery and prophylactic low molecular weight heparin (1 mg/kg/24 h) one day before and 10 days after LSG.

Nonetheless, he presented after 2 weeks with fever, vomiting, and abdominal pain. His examination revealed mild fever (38 °C), and tachycardia (100 bpm), with stable blood pressure. Abdominal examination showed mild midabdominal tenderness with slight distension. Triphasic pelviabdominal computed tomography (CT) revealed the presence of PMVT. The patient was admitted to the ICU. Bowel rest, IV fluids, broad-spectrum antibiotics, and therapeutic low molecular weight heparin (LMWH) 1 mg/kg/12 h were the treatment options.

Although the patient developed melena on the night of admission, we did not stop LMWH. The patient showed gradual improvement over the next 5 days, and he was discharged home one week after admission after ensuring adequate oral intake.

Two weeks later, the patient presented again with recurrent vomiting when some investigations were ordered, but the patient refused admission. Therefore, antiemetics were prescribed. However, the patient presented after 1 month with persistent vomiting with no response to the oral medications.

**Fig. 1:**
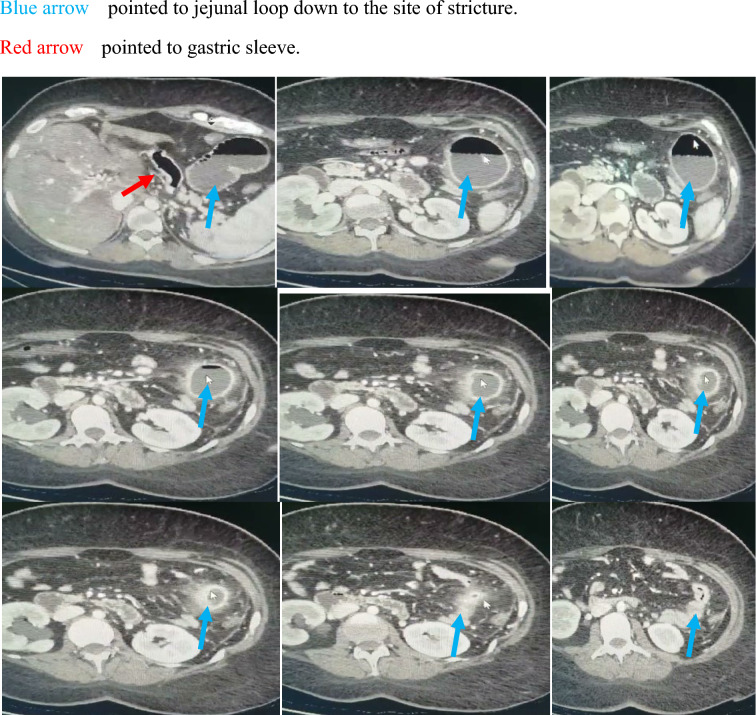
CT evaluation revealed, markedly dilated jejunal loop down to the site of jejunal stenosis (Color figure online)

Abdominal CT with oral and IV contrast was done, revealing the presence of jejunal stricture, Fig. 1. Laparoscopy was done, revealing the presence of narrowed jejunal segment about 20 cm from the Treitz ligament, which was managed by resection anastomosis.

### Case 2

A 49-year-old man with an initial BMI of 51 kg/m^2^ underwent LSG 1.5 years ago. His post-operative course was smooth and antithrombotic prophylaxis was performed, using mechanical compression during surgery and prophylactic low molecular weight heparin (1 mg/kg/24 h) one day before and two weeks after LSG.

However, the patient presented with abdominal pain only 1 week after LSG, and he was confirmed to have PMVT by triphasic pelviabdominal CT.

As the patient was clinically stable with no significant abdominal tenderness or leukocytosis, he was conservatively managed like the first one, with significant improvement in his symptoms. He was able to resume his oral intake on the fourth day, and he was discharged after 2 days.

**Fig. 2:**
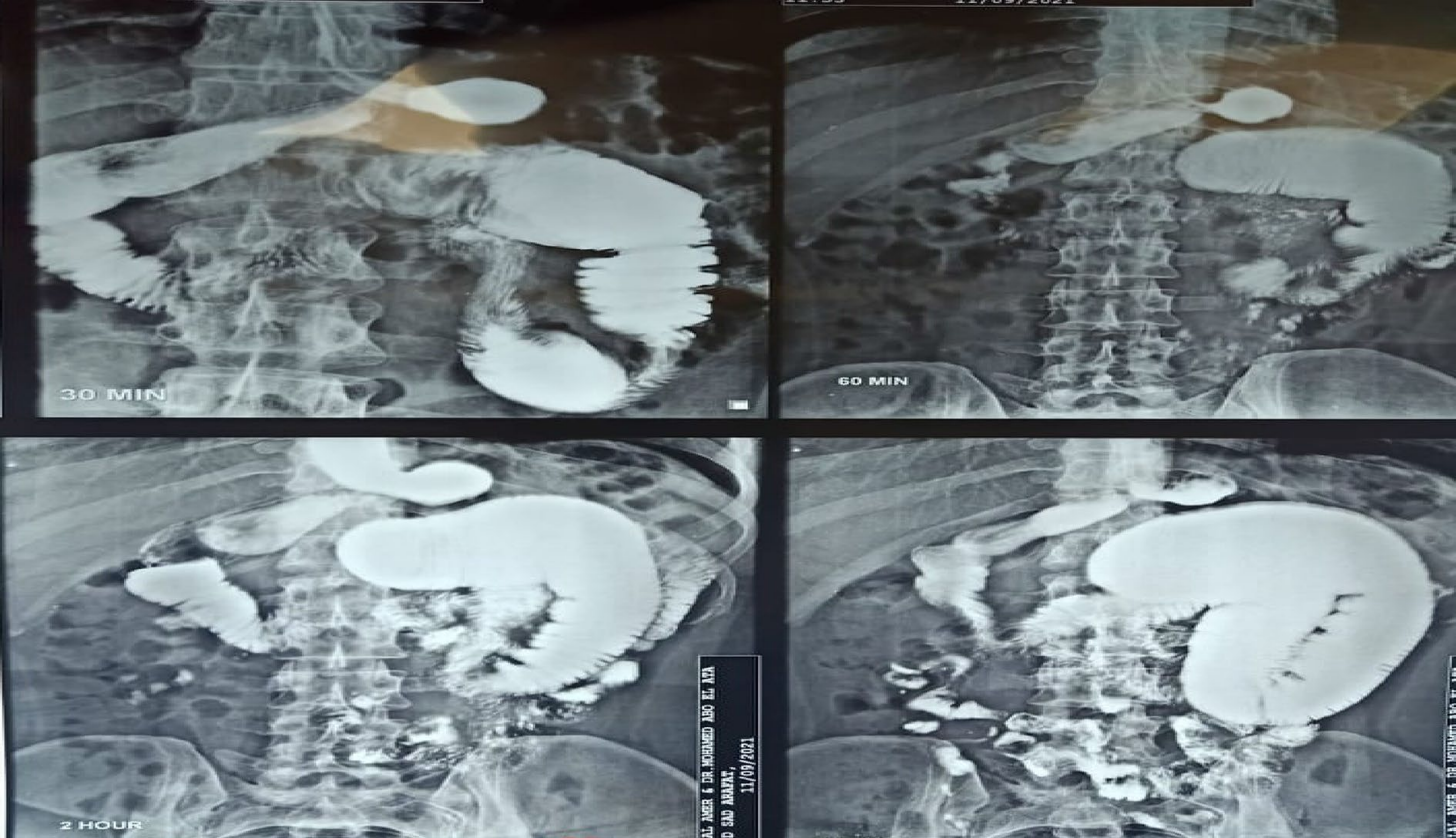
Barium study revealed marked dilatation of the proximal loop of the jejunum with delayed passage of the dye thorough the site of the stricture. Blue arrow pointed to jejunal loop down to the site of stricture. Red arrow pointed to the gastric tube

Two months later, the patient presented with persistent vomiting and upper abdominal distension. Upper gastrointestinal gastrografin series detected a focal segmental narrowing in the proximal jejunum, Fig. 2, and that was confirmed by abdominal CT. Laparoscopy was done, revealing the presence of narrowed jejunal segment about 50 cm from the Treitz ligament, which was managed by resection anastomosis.

### Case 3

A 35-year-old man whose BMI was 48.5 kg/m^2^ underwent LSG 6 months ago. The man was a heavy smoker. His post-operative course was smooth and antithrombotic prophylaxis was performed, using mechanical compression during surgery, and prophylactic low molecular weight heparin (1 mg/kg/24 h) one day before and two weeks after LSG.

He presented 6 weeks following the operation with severe periumbilical pain and bloody diarrhea. His examination revealed mild abdominal tenderness and stable vital signs. Laboratory workup revealed mild leukocytosis (WBCs 12.6 × 109/L on admission). Abdominal CT with oral and IV contrast was ordered, and it revealed PMVT.

The patient was admitted to the ICU, where he was commenced on IV fluids, antibiotics, and therapeutic LMWH, with close monitoring of his vital signs and frequent abdominal examination. There was a marvelous response to the conservative treatment, which was evident clinically and radiologically. The patient was able to resume oral intake after four days, and he was discharged one week after admission.

One month after discharge, he presented with persistent vomiting. Esophagogastroduodenoscopy revealed the presence of a hiatus hernia with mild duodenal dilatation. Barium follow-through was ordered, and it revealed the presence of proximal jejunal stricture with proximal dilatation and delayed passage of the dye beyond the stricture. CT with oral and IV contrast confirmed the diagnosis.

**Fig. 3:**
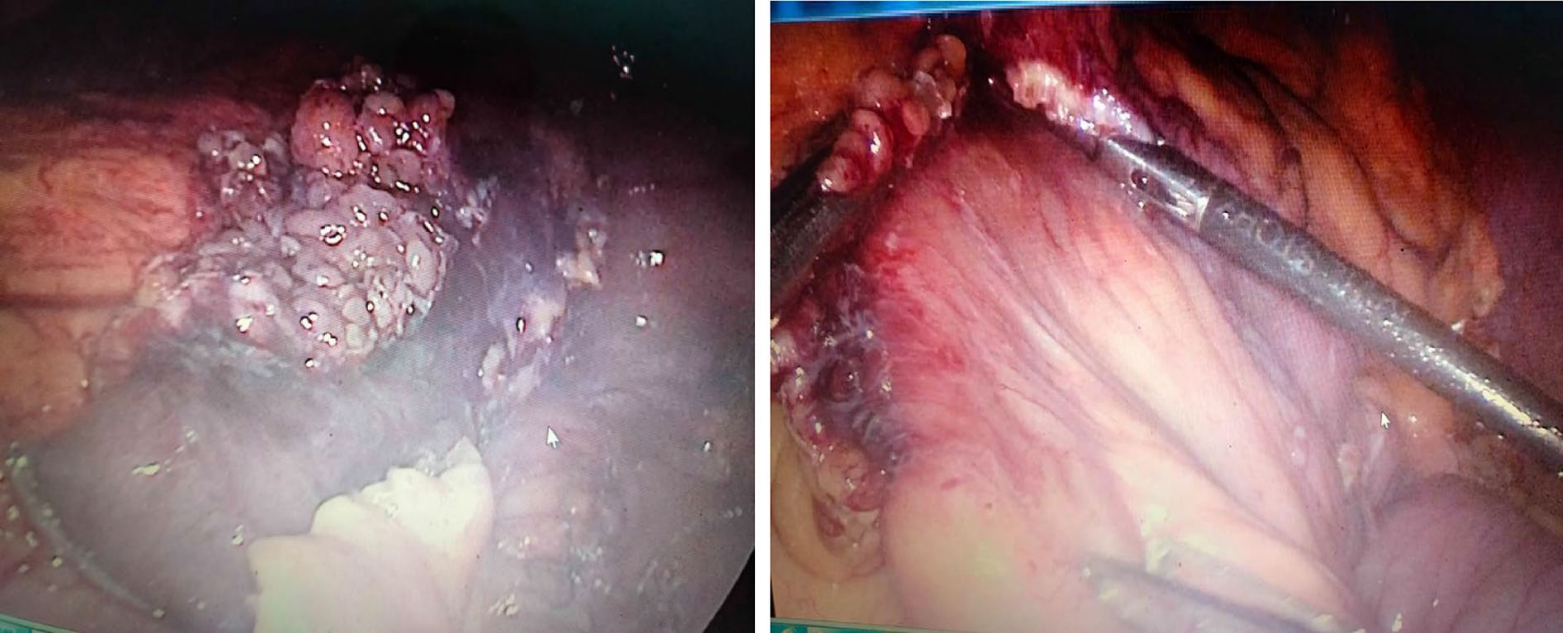
segment of the jejunum 30 cm from the DJ with omentum attached to it with dirty fat planes

**Fig. 4:**
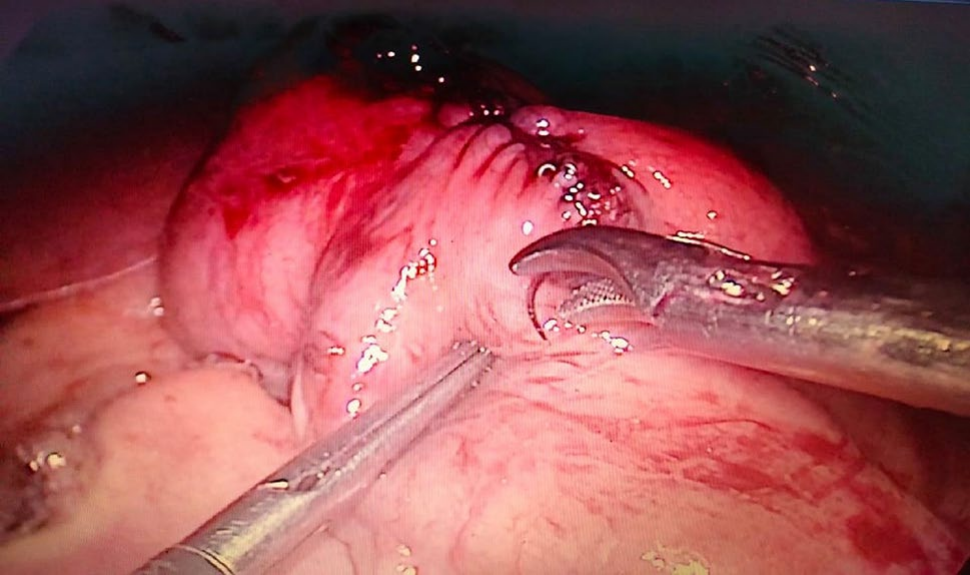
Side to side anastomosis after resection of the stenotic jejunal segment

Laparoscopic exploration was performed, and we detected a stenosed jejunal segment about 30 cm from the Treitz ligament with proximal bowel dilatation. This segment had omental attachments with dirty fat planes, Fig. 3. Resection anastomosis of this segment was done, Fig. 4. Followed by a crural repair for the hiatus hernia. The patient had an eventful course after the resection, and he was discharged home after five days.

## Discussion

Although bariatric surgery is effective in achieving weight loss and improvement of obesity-associated co-morbidities, it has its own morbidity including leakage, bleeding, gastroesophageal reflux, malnutrition, and cholelithiasis [15–21]. (Reviewer #4).

PMVT is a rare complication that is infrequently reported after LSG. Multiple factors are incriminated in the incidence of PMVT after bariatric surgery, including obesity itself, surgical trauma, pneumoperitoneum, and liver retraction. In addition, reverse Trendelenburg position and hypercapnia may predispose vasospasm and decrease portal blood flow leading to thrombosis [9].

Nonetheless, all of these factors are shared between different bariatric procedures and cannot explain the increased risk with LSG. That means that LSG has its own anatomical, physiological, and technical factors that may elicit this complication. These factors include the application of energy devices near the splenic vein, venous circulatory disturbances after the division of the short gastric vessels, and the inflammatory reaction following gastric division [22].

Although PMVT may be asymptomatic, the patient may have several manifestations like abdominal pain, fever, gastrointestinal bleeding, nausea, and vomiting. These symptoms usually develop within 1 month after the bariatric procedure. That should raise the surgeon's suspicion when the patient reports significant abdominal pain in the first month after LSG [23, 24]. The previously reported manifestations were detected in our patients, and all of them were presented within a month after the procedure, which agrees with the previous facts.

Laboratory investigations confirm the diagnosis is of limited efficacy, as leukocytosis and elevated C-reactive protein are present only in 20% and 10% of these cases. That also highlights the importance of clinical suspicion for early detection of this complication [9].

Although this complication could be diagnosed with duplex ultrasound, we preferred to depend on CT with contrast, as duplex is operator-dependent, and it would be difficult to assess the portal vasculature in such obese patients, especially with the presence of upper abdominal distension and ileus secondary to the existing ischemia [22].

One should highlight the importance of thromboprophylaxis after LSG. The duration of thromboprophylaxis is of crucial importance, as it was found that in most cases of PMVT after LSG received prophylaxis for less than 10 days. Therefore, it was recommended to extend the duration of prophylaxis up to four weeks after the operation [25].

The management of this condition depends on the patient clinical state. Most cases could be managed conservatively with hydration and anticoagulation when there is significant peritonitis or evidence of bowel wall ischemia on radiological investigations. Anticoagulation could be performed by unfractionated heparin, LMWH, vitamin K antagonists, or factor Xa inhibitors [23]. Nonetheless, bowel resection may be required in 20% of these cases, while splenectomy may be needed in 2% of them, according to a previous bariatric study [23].

In our series, the three described cases were successfully managed by anticoagulation and other conservative measures. However, they developed a rare complication in the short term, which was not previously discussed before in obese patients after LSG.

The incidence of small bowel strictures after PMVT is infrequently reported in the literature [13, 26, 27]. Additionally, a previous animal study reported the incidence of bowel stricture after deliberate embolization of a superior mesenteric arterial branch [28].

In 1995, Eugène et al. also reported the occurrence of small intestinal strictures in three patients with prior mesenteric venous thrombosis. The three cases required resection of the stenosed segment [13]. Antoch et al. reported similar findings in their case report that included two cases, and they were also managed by small bowel resection [29].

Bowel stricture following PMVT could be explained by the subsequent inflammation and scarring. This leads to bowel wall thickening and fibrosis of the muscular bowel layer, and that hinders the normal peristaltic wave, leading to obstruction [30].

As PMVT mainly affects the main portal and superior mesenteric veins [9], it is reasonable to find most of its consequences in the proximal small bowel (jejunum), as reported in our series.

Upper gastrointestinal series, along with abdominal CT with oral and IV contrast, greatly help in the diagnosis of this entity, especially if endoscopic evaluation of the upper GI tract revealed no significant anomalies.

As our patients had the manifestations of upper bowel obstruction, they were surgically managed by resection of the stenosed segment like in the previously mentioned studies.

The resected intestinal segment should be sent for histopathological diagnosis, as the patient may carry another pathology causing this entity like Crohn’s disease, tuberculosis, radiation enteritis, carcinoid infiltration, Behcet’s disease, nonsteroidal anti-inflammatory therapy, and lymphoma [30, 31]. The presence of mucosal ulcerations, submucosal edema, and fibrosis suggests the ischemic etiology [13].

All in all, we reported these three patients to raise the awareness of surgeons regarding this rare complication (intestinal stricture) that follows another rare one (PMVT) after bariatric surgery. That should help the bariatric community in the early diagnosis and management of such cases, especially since small bowel strictures are troublesome to be diagnosed by many physicians.

## Conclusion

Bariatric surgeons should be aware of the association between PMVT, following LSG, and ischemic bowel strictures. That should help in the rapid diagnosis of this rare and difficult entity. It is recommended to report such cases when encountered in order to specify the risk factors for this complication.
